# Reliability and Validity of Amharic Version of EORTC QLQ-C 30 Questionnaire among Gynecological Cancer Patients in Ethiopia

**DOI:** 10.1371/journal.pone.0157359

**Published:** 2016-06-15

**Authors:** Birhanu Abera Ayana, Shiferaw Negash, Lukman Yusuf, Wendemagegnhu Tigeneh, Demewoz Haile

**Affiliations:** 1 Department of Gynecology & Obstetrics, University of Gondar, Gondar, Ethiopia; 2 Department of Gynecology & Obstetrics, Addis Ababa University, Addis Ababa, Ethiopia; 3 Department of Clinical Oncology, Addis Ababa University, Addis Ababa, Ethiopia; 4 School of Public Health, Bahir Dar University, Bahir Dar, Ethiopia; Iranian Institute for Health Sciences Research, ACECR, ISLAMIC REPUBLIC OF IRAN

## Abstract

**Background:**

Cancer is a growing public health problem worldwide. The focus of cancer treatment, in addition to curation, is improving the quality of life (QOL). This study aimed to assess the reliability and validity of Amharic version of European Organization for Research and Treatment of Cancer Quality of Life Core Questionnaire (EORTC QLQ-C30) among gynecological cancer patients in Ethiopia.

**Methods:**

A facility-based cross-sectional study was conducted using the Amharic version of EORTC QLQ-C30 on 153 gynecological cancer patients in Tikur Anbassa Specialized Hospital (TASH), Addis Ababa, Ethiopia. Descriptive statistics, correlation analysis and multivariable linear regression were employed in statistical analysis.

**Results:**

The Amharic version of EORTC QLQ-C30 had a Cronbach’s α value of 0.81. The internal consistency for each domain of EORTC QLQ-C30 was also acceptable (Cronbach’s α >0.7) except for cognitive function domain (Cronbach’s α = 0.29). Stepwise multivariable linear regression analysis showed that emotional functioning (p<0.001), fatigue (p<0.001) and social functioning (p = 0.004) were the determinative scales of EORTC QLQ-C30 on global health status (GHS). The clinical validity test (Known group validity) showed that there were significant differences in score for twelve out of 15 domains, between surgery and radiation scheduled patients. All items of emotional function, role function, fatigue, and GHS meet the discriminate validity criterion.

**Conclusion:**

The Amharic version of EORTC QLQ-C30 found to be reliable and had an acceptable validity to assess the QOL for gynecological cancer patients. We recommend further work on the validity and responsiveness of the EORTC QLQ-C30 with stronger design.

## Introduction

Gynecological cancer describes any cancer of the female reproductive tract[1]. According to the global cancer statistics, gynecological cancers accounted for 19% of the 5.1 million estimated new cancer cases[2]. The gynecological cancer burden in developing countries is huge, primarily due to the high incidence and mortality of cervical cancer[3]. Cervical cancer is the most common gynecological malignancy in developing countries where organized screening programs barely exist[4, 5]. Besides cervical cancer, endometrial and ovarian cancers do contribute to some to the burden [6]. A 14 year review from the only radiotherapy center in Ethiopia showed gynecologic malignancies to be the most common cancer, accounting for about 36% of all cases and almost 47% among female patients[7].

In the care of patients with chronic and incurable disease like cancer, it is important to focus both on improving quality of life (QOL) and prolonging survival; hence, the extent to which treatment compromises QOL should also be taken into consideration[8]. Gynecologic cancers have a significant impact on QOL due to their effect on body image and sexual function[9]. A standard valid instrument is needed to assess QOL, so that, the result will be used to draw conclusions and to compare the results across similar studies. There are different types of tools used to assess QOL, and the choice of the instrument depends very much on the reason for measurement and the primary concepts of interest. When assessing QOL in a patient, disease-specific QOL scales are preferred because they are sensitive and are capable of detecting small but clinically significant changes in health[10].

In Ethiopia, there is no validated tool for assessment of QOL of cancer patients. The European Organization for Research and Treatment (EORTC) QLQ-C30 is one of QOL assessment instruments which is widely used worldwide. It consists of 30 questions, which are validated in several studies on various types of cancers[9, 11]. It is a copyrighted instrument owned by the EORTC and has been translated into 82 languages. However, the Amharic (Federal working language of Ethiopia) version of EORTCQLQ-C30 tool validity and reliability has not yet been assessed. Therefore this study aimed to investigate the validity and reliability of EORTCQLQ-C30 on gynecological cancer patients attending oncology unit of TASH, Addis Ababa, Ethiopia.

## Methodology

### Ethical consideration

Ethical clearance was obtained from the Institutional Review Board (IRB), College of Health sciences, Addis Ababa University (AAU), Ethiopia. Permission to use the EORTC QLQ-C30 questionnaire tool was secured from the EORTC. Patients were individually approached and informed about the purpose of the study; written consent was then obtained to confirm their willingness to participate. Patients were also assured that the completed questionnaires will not be stored in the patient’s clinical record and will remain confidential.

### Study setting

The study was conducted in the Departments of Gynecology and Obstetrics and Radiotherapy of TASH. TASH is a teaching hospital located in Addis Ababa, the capital city of Ethiopia, and it provides both teaching and clinical care services in different fields. It is also a major referral center from all corners of the country, especially for cancer patients. The Clinical Oncology Department is the only center in the country providing radiation therapy.

### Study design, sample size and sampling procedure

A facility based cross-sectional study was conducted from January 1 to June 30, 2014.The sample size (n) required for the study was calculated using the formula to estimate a single population using a coefficient of variation. The coefficient of variation for EORTC QLQ-C30 GHS showed a CV of 0.30 which was considered for sample size calculation[12]: In this study we assumed, 95% confidence level, 5% of absolute precision, the final sample size was calculated based on the formula $\text{n}=\left[\frac{{\left(\text{z}\frac{\alpha }{2}\right)}^{2}(\text{CV})2}{{\text{d}}^{2}}\right]$$\text{n}=\left[\frac{{\left(1.96\right)}^{2}(0.3)2}{0.05^{2}}\right]=138.29=139$

By considering the 10% non-response rate, the total sample size was 153 gynaecological cancer patients. All patients who came to the specified departments during the study period and fulfilled the inclusion criteria were included in the study until the sample size was achieved. Totally 140 gynecological cancer patients were included in the analysis. A total of 13 patients were excluded from the analysis because they were not interested to be included in the study. This study had included patients with age 18 years and older who are treated for the first time for gynaecological cancer. Patients who had previously received cancer treatment were excluded from the study, as were, patients with psychiatric disorders, communication disorders, other severe medical illnesses, coexisting malignancies, and positive HIV sero-status.

### Data Collection Instrument

The Amharic version of EORTC QOL-C30 is composed of 30 questions. Of the 30 items, 24 are organized into nine scales: Physical functioning (5 items; questions from 1 to 5), Role functioning(2 items; question 6 and 7), Emotional functioning(4 items; questions from 21 to 24), Cognitive functioning(2 items; question 20 and 25), Social functioning(2 items; question 26 and 27), GHS/quality of life(2 items; question 29 and 30), Fatigue(3 items; questions 10, 12 and 18), Nausea and vomiting(2 items; question 14 and 15), and Pain(2 items; question 9 and 19) and 6 single items assessing financial impact and various physical symptoms such as dyspnea, insomnia, appetite loss, constipation and diarrhea(questions 28, 8, 11, 13, 16 and 17 respectively[13]).

### Scoring procedures

The row scores were transformed to 0 to 100 based on the recommended formulas in the scoring manual for each EORTC QLQ-C30 component [14]. A high score for a functional scale represents a high/healthy level of functioning whereas a high score for a symptom scale or item represents a high level of symptomatology or problems [13]. The translation of the EORTC QLQ-C30 into Amharic version was made by another previous project. This study had received the translated Amharic EORTC QLQ-C30 from EORTC with grant to use for the proposed study.

### Statistical analysis

Data was checked for completeness and consistency, cleaned, coded, and entered to SPSS version 20 windows. Descriptive statistics and independent t-test were employed.

The internal consistency of EORTCQLQ-C30 measured by the Cronbach’s α coefficient for each domain. The Cronbach’s α value higher than 0.7 is generally considered to be satisfactory[14]. Stepwise multivariable linear regression model was fitted to identify the most determinative components of EORTCQLQ-C30 against Global health status (Criterion validity). The standardized regression coefficient was reported with p values p< 0.05 considered statistically significant. Multi-trait scaling analysis was used to test the convergent and item discriminant validity of the EORTC QLQ-C30. Convergent validity was revealed if the item domain correlation was ≥ 0.40[15, 16], while the requirements for discriminant validity were satisfied if the value of correlation coefficients between the item and its own domain was higher than other domains[16]. Known-groups validity was evaluated by comparing groups with a clinically evident difference using an independent t-test.

## Result

### Sample Characteristics

A total of 140 patients in whom a diagnosis of a single entity of gynaecologic cancer is suspected or confirmed were included in the analysis. The mean (SD) age for the participants was 53.06(12.45) years. Of the total 140 participants 68.5% have never gone to school. With respect to marital status 52.9% of participants are currently on marriage while the rest of the study participants were not (i.e. divorced, widowed or single). Majority of participants come from outside Addis Ababa. Most of the participants (62.1%) were housewives (**Table 1**)

**Table 1:** Socio-demographic characteristics of gynaecological cancer patients attending treatment at TASH, Addis Ababa, and Ethiopia 2014.

Variables		Frequency	Percentages
Age	< 40 years	18	12.9
	40–49 years	36	25.7
	50–59 years	42	30.0
	60–69 years	25	17.9
	≥70 years	19	13.6
Marital status	Currently on marriage	74	52.9
	Currently not on marriage	66	47.1
Educational status	Never go to school	96	68.6
	Primary	24	17.1
	Secondary	11	7.9
	12+	9	6.4
Parity order	0	6	4.3
	1–4	47	33.6
	5–9	70	50.0
	≥10	17	12.1
Address	Out of Addis Ababa	101	72.1
	Addis Ababa	39	27.9
Ethic group	Amhara	64	45.7
	Oromo	46	32.9
	Tigre	14	10.0
	Gurage	11	7.9
	Others (keficho, Hadiya, Siltea)	5	3.6
Religion	Orthodox Christian	96	68.6
	Muslim	28	20.0
	Protestant	16	11.4
Occupation	House wife	87	62.1
	Self employed	20	14.3
	Farmer	16	11.4

The internal consistency of the Amharic version of EORTC QLQ-C30 had a Cronbach’s α value of 0.81. All of the domains have an acceptable internal consistency except for cognitive function domain with Cronbach’s α = 0.29 (Table 2).

**Table 2:** Internal consistency of EORTC QLQ-C30 questionnaire on each domain among gynecological cancer patients attending TASH, Addis Ababa, Ethiopia, 2014.

Domain (sub-scales/items)	Internal consistency (Cronbach’s coefficient)
**Overall EORTCQLQ C 30**	0.81
**Functional domain**	0.79
Physical function	0.83
Emotional function	0.93
Cognitive function	0.29
Global health status	0.92
Social function	0.82
**Symptom domain**	0.81
Fatigue	0.89
Pain	0.73
Nausea and vomiting	0.75
Dyspnoea	Single item
Role function	Single item
Insomnia	Single item
Appetite loss	Single item
Constipation	Single item
Diarrhea	Single item
Financial difficulties	Single item

Most of the correlation between inter domain scales of EORTC QLQC-30 was found statistically significant. Insomnia, loss of appetite, pain, fatigue, financial difficulties, and constipation were significantly negatively correlated with all of the functional domain components and GHS (p<0.001). Nausea and vomiting were negatively correlated with all functional domains (physical, social, emotional, and role function) and GHS except cognitive function (p<0.05). Diarrhea was not significantly correlated with any of the functional domain and GHS, p>0.05 (Table 3).

**Table 3:** Correlation among EORTC QLQ-C30 components for measuring QOL among gynecological cancer patients attending TASH, 2014.

Correlation		Social Function	Cognitive Function	Global Health score	Emotional function	Physical function	Role function
Dyspnoea	r	-0.15	-0.15	-0.05	-0.115	-0.16	-0.109
	P-value	0.09	0.08	0.53	0.18	0.06	0.200
Insomnia	r	-0.41	-0.38	-0.42	-0.294	-0.56	-0.536
	P-value	<0.001	<0.001	<0.001	<0.001	<0.001	<0.001
Appetite loss	r	-0.41	-0.35	-0.50	-0.48	-0.47	-0.534
	P-value	<0.001	<0.001	<0.001	<0.001	<0.001	<0.001
constipation	r	-0.56	-0.37	-0.50	-0.400	-0.404	-0.549
	P-value	<0.001	<0.001	<0.001	<0.001	<0.001	<0.001
Diarrhea	r	0.12	0.07	0.060	0.033	-0.04	-0.016
	P-value	0.15	0.38	0.479	0.69	0.66	0.851
Financial difficulties	r	-0.72	-0.29	-0.452	-0.46	-0.385	-0.502
	P-value	<0.001	<0.001	<0.001	<0.001	<0.001	<0.001
Pain	r	-0.59	-0.40	-0.603	-0.50	-0.66	-0.087
	P-value	<0.001	<0.001	<0.001	<0.001	0.001	0.305
Nausea and Vomiting	r	-0.18	-0.104	-0.172	-0.234	-0.182	-0.672
	P-value	0.03	0.22	0.042	0.005	0.031	<0.001
Fatigue	r	-0.617	-0.377	-0.614	-0.499	-0.752	-0.758
	P-value	<0.001	<0.001	<0.001	<0.001	<0.001	<0.001

### Criterion validity

All the components of EORTC QLQ-C30 were modeled against the GHS score via stepwise linear regression model. The results showed that emotional functioning (p<0.001), fatigue (p<0.001), and social functioning (p = 0.004) were the determinative scales of QLQ-C30 on GHS (Table 4).

**Table 4:** Stepwise multivariable linear regression model to evaluate validity EORTC QLQC-30 components against GHS.

EORTC QLQC-30 components	Standardized Beta	T	P value
Fatigue	-0.321	-4.05	<0.001
Emotional function	0.295	4.04	<0.001
Social function	0.236	2.93	0.004

### Clinical validity (known-groups validity)

Known-groups validity was evaluated based on different treatment types. This study assumed that patients receiving surgery would report a better QOL as compared to patients receiving radiation. Generally speaking, cervical cancer patients at an earlier stage can be treated by surgery and those at a late stage are treated by radiation. Thus, we selected two subgroups among cervical cancer patients, a surgery group (56 cases) and a radiation group (84 cases), and compared the mean QOL scores between the two groups by t-tests. There were significant differences for twelve out of 15 domains (Table 5).

**Table 5:** Clinical validity of the simplified Amharic version of EORTC QLQ-C30, TASH, Addis Ababa, Ethiopia, 2014.

Variables	Operation Mean(±SD)	Radiation Mean(±SD)	p-value
**Global Health status**	49.99±24.77	34.92±22.24	0.001
**Physical function**	75.12±17.78	58.65±23.14	<0.001
**Emotional function**	65.03±29.36	49.12±29.41	0.002
**Role function**	65.48±29.96	39.88±34.71	<0.001
**Social function**	55.95±34.15	33.14±27.19	<0.001
**Cognitive function**	92.85±13.05	85.12±20.86	0.015
**Fatigue**	39.68±24.88	63.49±26.92	<0.001
**Nausea & vomiting**	11.31±19.62	6.15±16.52	0.096
**Pain**	37.20±25.02	67.06±26.38	<0.001
**Dyspnea**	8.33±19.33	5.56±17.03	0.372
**Insomnia**	23.81±27.50	44.44±34.84	<0.001
**Diarrhea**	1.79±7.57	0.79±7.27	0.438
**Financial difficulties**	50.00±37.07	74.60±24.61	<0.001
**Constipation**	26.78±35.06	55.95±37.37	<0.001
**Appetite loss**	36.31±37.75	49.21±35.28	0.041

The convergent validity, which is measured by correlations between the item and its own domain, was not acceptable for most of the domains, except for fatigue, role function, and GHS. The physical function domain did not meet discriminant validity criterion except in item 3. Physical function items correlated more strongly with fatigue domain than its own domain. All items of emotional function, role function, fatigue, and GHS meets the discriminant validity criterion. Item 9, 19, 25, and 26 did not meet the discriminant validity criterion (Table 6).

**Table 6:** Convergent and Item discriminant validity of EORTC QLQ-C30 among gynecological cancer patients, TASH, Addis Ababa, Ethiopia, 2014.

Items		GHS^@^	PF^#^	EF^$^	RF^&^	SF^ø^	CF “	Fatigue	NV^	Pain
Strenuous activity	r	-0.026*	-0.323*	-0.128	-0.336**	-0.264**	-0.099	0.387**	0.084	0.029**
Long walk	r	-0.268**	-0.295**	-0.157	-0.298**	-0.228**	-0.085	0.351**	0.033	0.256**
Short walk	r	-0.227**	-0.373**	-0.036	-0.223**	-0.175*	-0.201*	0.352**	0.029	0.255**
Stay in bed/chair	r	-0.222**	-0.289**	-0.068	-0.123	-0.200*	-0.039	0.305**	0.013	0.201*
Needed help: eating/dressing/washing	r	-0.212*	-0.242**	-0.133	-0.185*	-0.181*	-0.095	0.256**	0.032	0.176*
Limited work	r	-0.298**	-0.314**	0-.142	-0.461**	-0.306**	-0.137	0.419**	-0.026	0.296**
Feel tense	r	-0.179*	-0.212*	-0.296**	-0.189*	-0.214*	0.090	0.226**	0.071	0.181*
Worried	r	-0.176*	-0.174*	0-.308**	-0.149	-0.254**	0.040	0.227**	0.040	0.230**
Feel irritable	r	-0.208*	-0.191*	-0.279**	-0.194*	-0.177*	0.001	0.266**	0.008	0.216*
Feel depressed	r	-0.173*	-0.059	-0.332**	-0.132	-0.141	0.123	0.127	0.021	0.116
Concentration	r	-0.227**	-0.309**	-0.160	-0.260**	-0.329**	-0.209*	0.343**	-0.006	0.299**
Remembering	r	0.066	0.017	0.101	0.060	-0.036	-0.179*	0.011	0.017	-0.014
Family life	r	-0.235**	-0.377**	-0.271**	-0.269**	-0.343**	-0.126	0.294**	0.045	0.263**
Social life	r	-0.343**	-0.300**	-0.267**	-0.262**	-0.375**	-0.192*	0.361**	0.128	0.280**
Need rest	r	-0.266**	-0.396**	-0.088	-0.362**	-0.240**	-0.125	0.443**	0.039	0.302**
Felt week	r	-0.236**	-0.324**	-0.098	-0.299**	-0.319**	-0.205*	0.407**	0.030	0.279**
Tired	r	-0.222**	-0.280**	-0.173*	-0.317**	-0.286**	-0.185*	0.422**	0.069	0.321**
Nausea	r	0.018	-0.043	0.037	0.024	-0.086	0.244**	0.053	0.292**	0.007
Vomiting	r	-0.005	0.033	-0.020	0.072	-0.118	0.141	0.023	0.487**	-0.024
Pain	r	-0.203*	-0.314**	-0.128	-0.298**	-0.306**	-0.082	0.382**	0.062	0.333**
Pain interfere with daily activities	r	-0.282**	-0.332**	-0.130	-0.387**	-0.317**	-0.176*	0.420**	-0.078	0.350**
Overall Health condition	r	0.445**	0.304**	0.180*	0.268**	0.320**	0.071	-0.35**	0-.020	-0.29**
Overall quality of life	**r**	0.410**	0.227**	0.194*	.256**	0.243**	0.026	-0.26**	0.031	-0.22**

@ Global health score

^&^ Role function

^ø^social function

“cognitive function

^ Nausea and vomiting

# Physical function

$ Emotional function

*significant at p value 0.05

** significant at p value 0.001

## Discussion

Of the 140 participants interviewed, only 5% were able to answer question number ‘‘7” of the EORTC QLQ C-30 which assesses role function together with number “6”. The remaining patients stated that they had never been involved with such activities even before their illness. In a similar study done in Tunisia,48% of the participants only answered this question [17]. This may possibly be explained by the difference in socioeconomic as well as psychosocial and cultural makeup of the study population.

The internal consistency of the Amharic version of EORTC QLQ-C30 was acceptable (Cronbach’s α>0.7). The individual domains with the exception of the cognitive domain (cronbach α = 0.29, were in the acceptable ranges of internal consistency.). Similar findings were reported from other studies conducted in different countries [18–20]. The low Cronbach’s α value in cognitive function described in our study subjects was similar to the reported findings by different studies [21–24]. This means that the items for constructing cognitive domain (item 20 and item 25) are not correlated. In fact a patient might not concentrate well due to pain or fatigue, which again in turn affect the memory[25].

The criterion validity analysis in our study showed that emotional functioning, fatigue, and social functioning were the determinative scales of QLQ-C30 on GHS. This implies that the gynecological cancer patients in Ethiopia rate their QOL based on their emotional functioning, fatigue and social functioning. Similarly findings reported from Turkey showed the most determinative sub-scales of QLQ-C30 on GHS were emotional functioning, fatigue, role functioning, and appetite loss[26].

The domains fatigue, role function, GHS meet the convergent validity criteria (r≥ 0.4). However, the physical function items correlated more strongly with fatigue domain than its own domain. A study from Morocco reported that all items exceeded the 0.4 criterion for convergent validity on all scales. The item discriminate validity which was acceptable for all items except item 3, was similar to the Moroccan study, which also demonstrated that fatigue items were highly correlated with the physical functioning scale[21]. Similarly, a study from Netherlands on Turkish and Moroccan respondents discovered high correlation between fatigue items and physical function scale[22]. This might be due to patients’ understanding how the items are categorized. Patients might respond to physical function items based on their feelings of tiredness, weakness, and need of rest which are the items for fatigue.

Clinical validity test was done only on cervical cancer patients based on the intended treatment type. The assumption was that those patients receiving surgery would have better QOL scores as compared to those receiving radiation. In this study, a statistically significant better score was achieved among the group who are scheduled for surgery in all domains except in nausea and vomiting, diarrhea, dyspnea, and cognitive function. The absence of statistically significant difference in, diarrhea, dyspnea nausea and vomiting / between operated and irradiated patients could be these items were not common and not dependent on stage of cervical cancer patients. Similarly the absence of statistically significant difference between the two treatment groups in cognitive function might be due to lower reliability of the tool as indicated by the low Cronbach’s α value. Similar finding was reported from the Chinese study, where clinical validity was tested with a similar logic. That study yielded a similar outcome, with the surgery group achieving better scores in 9 out of 15 domains [24].

This study had its own of limitations. First this study was a cross-sectional study and we were not able to determine, test-retest reliability and responsiveness of EORTC QLQ-C30 over time. Furthermore the external convergent validity; the gold standard test to assess validity, was not tested in this study due to unavailability of other validated QOL assessment tool in Ethiopia.

## Conclusion

The Amharic version of EORTC QLQ-C30 was found out to be reliable and had an acceptable validity for assessing QOL of gynecological cancer patients in Ethiopia. However, further work with strong design on the validity of some domains and on the responsiveness of the EORTC QLQ-C30 is recommended.

## Supporting Information

S1 Dataset The dataset from which the manuscript is produced. (SAV)
